# Dissection of Functional Domains of Orc1-2, the Archaeal Global DNA Damage-Responsive Regulator

**DOI:** 10.3390/ijms232314609

**Published:** 2022-11-23

**Authors:** Xiaotong Liu, Mengmeng Sun, Ruyi Xu, Yulong Shen, Qihong Huang, Xu Feng, Qunxin She

**Affiliations:** 1 CRISPR and Archaea Biology Research Center, Microbial Technology Institute and State Key Laboratory of Microbial Technology, Shandong University, 72 Binhai Road, Jimo, Qingdao 266237, China; 2 Archaea Centre, Department of Biology, University of Copenhagen, Ole Maaløes Vej 5, DK-2200 Copenhagen, Denmark

**Keywords:** origin recognition complex, DNA damage response, AAA+ ATPase, winged-helix domain, archaea, global regulator

## Abstract

Orc1-2 is a non-initiator ortholog of archaeal/eukaryotic Orc1 proteins, which functions as a global regulator in DNA damage-responsive (DDR) expression. As for Orc1 initiators, the DDR regulator harbors an AAA+ ATPase domain, an Initiator-Specific Motif (ISM) and a winged-helix (wH) DNA-binding domain, which are also organized in a similar fashion. To investigate how Orc1-2 mediates the DDR regulation, the *orc1-2* mutants inactivating each of these functional domains were constructed with *Saccharolobus islandicus* and genetically characterized. We found that disruption of each functional domain completely abolished the DDR regulation in these *orc1-2* mutants. Strikingly, inactivation of ATP hydrolysis of Orc1-2 rendered an inviable mutant. However, the cell lethality can be suppressed by the deficiency of the DNA binding in the same protein, and it occurs independent of any DNA damage signal. Mutant Orc1-2 proteins were then obtained and investigated for DNA-binding in vitro. This revealed that both the AAA+ ATPase and the wH domains are involved in DNA-binding, where ISM and R381R383 in wH are responsible for specific DNA binding. We further show that Orc1-2 regulation occurs in two distinct steps: (a) eliciting cell division inhibition at a low Orc1-2 content, and this regulation is switched on by ATP binding and turned off by ATP hydrolysis; any failure in turning off the regulation leads to growth inhibition and cell death; (b) activation of the expression of DDR gene encoding DNA repair proteins at an elevated level of Orc1-2.

## 1. Introduction

The organisms of all three domains of life have evolved a sophisticated signal transduction network known as DNA damage response (DDR) regulation to safeguard genomic integrity, but the main regulators responsible for the regulation are not evolutionarily related. Bacteria primarily employ the proteolysis of LexA, a global transcriptional repressor of bacterial SOS response, to liberate the expression of a large number of genes involved in cell cycle arrest and DNA repair [1]. Eukaryotes have adopted a cascade of phosphorylation on key factors that are mediated by three related kinases (DNA-PK, ATM, and ATR) to trigger a global response upon different types of DNA damage [2]. For archaea, however, it has been demonstrated that Orc1-2, an ortholog of the archaeal/eukaryotic origin recognition complex (Orc) proteins, plays a central role in DDR regulation [3]. However, the involved mechanism for Orc1-2 regulation in archaea remains to be studied.

Orc1-2 proteins are evolutionally related to the archaeal/eukaryotic origin recognition complex, Orc1, and to the cell division control protein 6, Cdc6 [4,5]. Crenarchaea such as those belong to the Sulfolobales order code for three Orc1 proteins. Their encoding genes have been functionally characterized in *Saccharolobus islandicus* (formerly *Sulfolobus islandicus*) [6], an extremely thermophilic crenarchaeon isolated from a terrestrial hot spring in Iceland [7], and this has revealed that Orc1-1 and Orc1-3 function as replication initiator protein (termed initiator Orc1), whereas Orc1-2 does not (named non-initiator Orc1) [8]. To date, several archaeal initiator Orc1 proteins have been characterized for their interactions with the archaeal origins of DNA replication, including those from *Pyrobaculum aerophilum* [9], *Methanothermobacter thermoautotrophicus* [10,11], *Pyrococcus abyssi* [12,13], *Saccharolobus solfataricus* [14,15,16], *Archaeoglobus fulgidus* [17], *Aeropyrum pernix* [18], *Thermoplasma acidophilum* [19], *Sulfolobus acidocaldarius* [20], *S. islandicus* [8], *Picrophilus torridus* [21], and *Haloferax volcanii* [22]. These proteins bind specifically to a conserved DNA sequence motif, termed origin recognition boxes (ORBs), present in their cognate origins of replication [11,14,23]. These archaeal Orc1 proteins share the same modular organization of structural domains: an AAA^+^ ATPase domain at their N-termini and a winged helix (wH) DNA-binding domain at the C-termini [9,18,24,25], and both domains contribute to the specific binding of these replication initiators to ORBs at the replication origins [16,24,25,26]. Thus, it is very interesting to investigate how the structural domains of Orc1-2 function in DDR regulation, including the onset of DNA damage response as well as offsetting the process.

In this work, *S. islandicus* REY15A was employed as the archaeal model [27] to functionally characterize Orc1-2. The endogenous CRISPR-based DNA mutagenesis was employed to generate Orc1-2 derivatives carrying mutations in highly conserved amino acids in the ATPase and DNA-binding domains. Genetic and biochemical characterization of the mutants revealed that both the N-terminal ATPase domain and the C-terminal wH domain are essential for Orc1-2-mediated DDR regulation, and ATP hydrolysis is required for offsetting the DDR process.

## 2. Results

### 2.1. Structural Modelling Revealed Key Residues in Orc1-2 Responsible for DNA Binding

**Figure 1:**
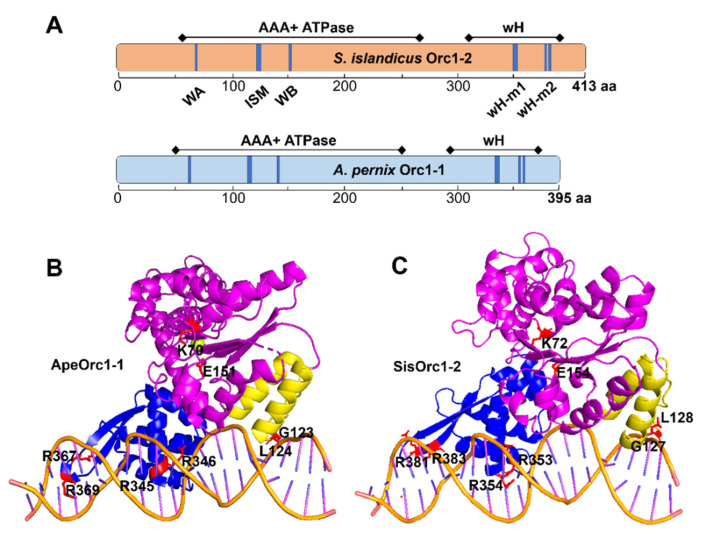
Structural modeling reveals DNA binding motifs in the wH domain of Orc1-2. (**A**) Schematic of domain organization of the *S. islandicus* Orc1-2 and *A. pernix* Orc1-1. Positions of conserved motifs are highlighted in blue. (**B**) Crystal structure determined for the *A. pernix* ORC1 protein in complex with DNA (PDB: 2V1U). The AAA+ ATPase domain is shown in purple and ISM is colored dark yellow, whereas the wH domain is in blue. Residues chosen for substitution are shown as red sticks. (**C**) Simulated structure of Orc1-2 ADP on DNA generated with PyMOL using the structure in (**B**) as the template. Conserved motifs are highlighted in red in each functional domain, which were subjected to mutagenesis, as specified in Table 1.

Archaeal Orc1 initiators have three structural domains, i.e., an N-terminal AAA^+^ ATPase domain, an ISM, and a C-terminal winged helix (wH) [16,24,25,26], all of which are present in Orc1-2, the non-initiator (Figure 1A). Sequence alignments of a few Orc1 proteins of both initiators and non-initiators revealed that key amino acid residues in these structural domains are conserved in both types of archaeal Orc1 proteins, including Walker A and B motifs in the ATPase domain, which are responsible for ATP binding and hydrolysis, respectively, as demonstrated for the *S. islandicus* Orc1-1 protein [28,29,30,31], and the ISM. However, differences were also observed: the four arginine residues in the wH domain of Orc1 initiators, which interact with the ORB motif in the archaeal origins of chromosome replication, do not occur at the corresponding positions in Orc1 non-initiators (Figure S1).

To identify which residues could be involved in protein–DNA interaction in Orc1-2, structural modelling was conducted for *S. islandicus* Orc1-2 with the structures of the DNA-bound form of three Orc1 initiators as templates, including *A. pernix* Orc1 and *S. solfataricus* Orc1-1 and Orc1-3. We found 4 arginine residues (R353 and R354; R381 and R383) located in close proximity to DNA in the model, in analogy to the pairs of R345 and R346; R367 and R369 in ApeOrc1-1 (Figure 1B). To this end, it was very interesting to investigate how Orc1-2 utilizes an architecture of the initiator domain organization to function as a global transcriptional regulator to regulate hundreds of DDR genes.

### 2.2. Inactivation of ATP Hydrolysis of Orc1-2 Yielded Cell Death in S. islandicus

To functionally characterize the conserved domains in Orc1-2, genome editing plasmids were constructed for generating gene truncations at the C- or N-terminus (N or C) and point substitutions of the conserved amino acids in the conserved domains of ATPase, ISM and wH (highlighted in Figure 1) for the *S. islandicus orc1-2* gene (Table S1). Gene editing experiments revealed that all designed mutations were successfully obtained, except for *orc1-2^E154A^*, which inactivates the ATP hydrolysis of the AAA+ ATPase at the Walker B domain (Table 1) [8]. These results indicated that inactivation of the ATP hydrolysis of Orc1-2 could be lethal to this archaeon and the Orc1-2E154A-driven cell death may not rely on the transduction of any DNA damage signal.

To gain an understanding of the Orc1-2E154A-driven cell death, we attempted to generate the E154A substitution using wH, the DNA-binding deficient mutant (see Section 2.5) to test if the cell lethality could be DNA-binding dependent. We found that double domain mutants of WBwH were readily obtained with *S. islandicus*. Therefore, the wH mutation can suppress the cell lethality phenotype of *orc1-2^E154A^*. Thus, we went further to study the functions of Orc1-2 domains in the archaeal DDR regulation.

### 2.3. Each Functional Domain of Orc1-2 Is Essential for DNA Damage Tolerance of S. islandicus

All constructed *orc1-2* mutants were physiologically characterized. They showed very similar patterns of growth under ambient conditions; however, the addition of 2 µM NQO, a DNA damaging agent, to the growth medium completely inhibited the growth of all *orc1-2* mutants inactivated for individual functional modules, including WA, ISM, and wH (Table 1, Figure S2A,B). These results indicated that all conserved functional modules are essential for mediating the NQO resistance. Flow cytometry of each sample suggested that most of cells of the WT strain have recovered from the DNA damage treatment at 24 h, whereas the DNA-less cells dominate in the NQO-treated cultures of the *orc1-2* deletion mutant. The functional deficiency in each domain basically yields the phenotype of Orc1-2 deficiency observed with the gene deletion mutant (Table 1, Figure S2C). Thus, both domains and the conserved amino acids therein are essential for the function of Orc1-2 in mediating DNA damage resistance in this archaeon.

### 2.4. All Orc1-2 Functional Modules Are Important for Regulated Expression of DDR genes in S. islandicus

In a previous work, we found that Orc1-2 regulates a large category of genes, including both up-regulated genes and down-regulated ones [3]. Representative genes of both categories were chosen for testing how these Orc1-2 mutations could affect their expression under ambient conditions and in the presence of NQO. Up-regulated genes included *orc1-2* (SiRe_1231), *tfb3* (SiRe_1717), *upsA* (SiRe_1881), and *cedA1* (SiRe_1316), which either encode the main DDR regulators or the proteins responsible for DNA transfer. Down-regulated genes included *orc1-1* (SiRe_1740), *orc1-3* (SiRe_0002), *vps4* (SiRe_1175), *ESCRT-III-1* (SiRe_1550), and *ESCRT-III-3* (SiRe_1200), all of which function in cell cycle progression. Their expression was examined by analysis of encoded proteins by Western blot analysis, reporter gene assay, or RT-qPCR.

**Figure 2:**
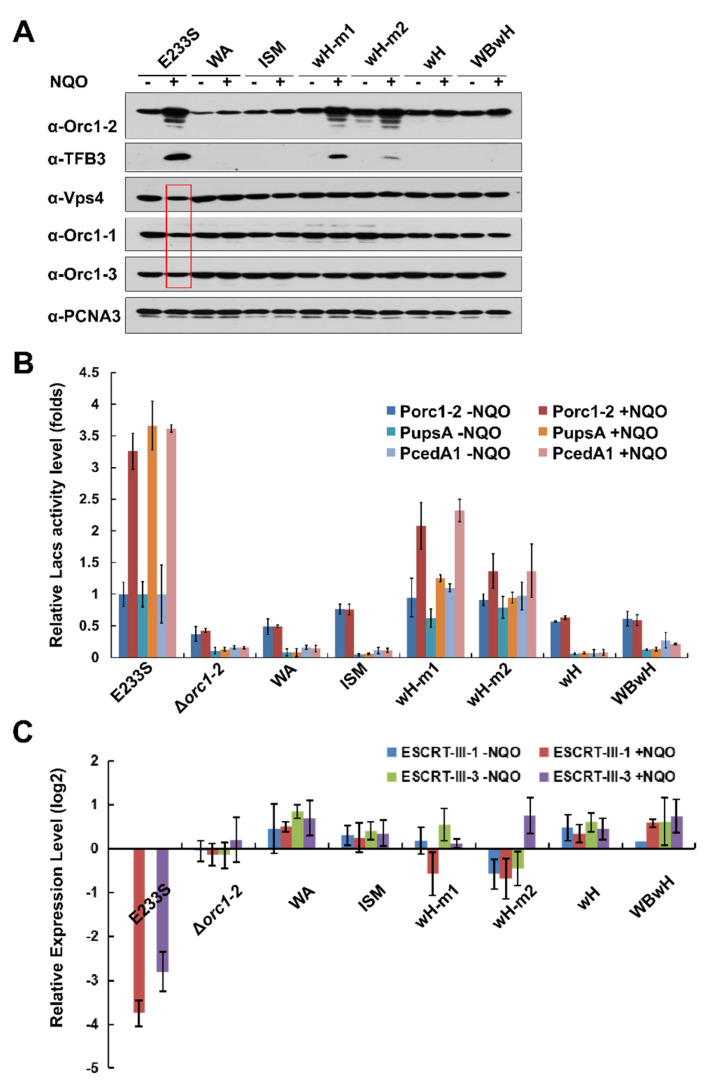
NQO-responsive expression of DDR genes in different *orc1-2* mutants. (**A**) Western blot analysis of selected DDR genes. PCNA3 (one of the subunits of the replication clamp) was used as the loading reference. E233S, WT strain; WA, K72A at Walker A motif; ISM, G127DL128D at the initiator specific motif (ISM); wH-m1, R353AR354A at the wH domain; wH-m2, R381AR383A at the wH domain; wH, quadruple substitutions at wH domain; WBwH, pentuple substitutions at Walker B motif and wH domain. (**B**) Reporter gene assay of three highly up-regulated genes (*orc1-2*, *upsA* and *cedA1*). Cell extracts prepared from cultures of E233S and *orc1-2* mutants carrying reporter gene plasmid of each promoter were assayed for ꞵ-glycosidase activity. (**C**) RT-qPCR of *ESCRT-III-1* and *ESCRT-III-3*. The expression level of 16SRNA was used to normalize the data. *Saccharolobus* cells were grown in the presence or absence of 2 μM NQO (indicated as +NQO and −NQO, respectively) for 6 h. Cell mass was collected from each culture with which cell extracts were prepared for Western blot analyses and for reporter gene assay, whereas total RNAs were prepared for RT-qPCR analysis.

First, the protein stability of mutated as well as the wild-type Orc1-2 proteins was assessed by Western blot analysis with cell extracts prepared from the corresponding *S. islandicus* strains. As shown in Figure 2A, WT and mutated Orc1-2 proteins of the predicted sizes were identified in individual mutants, except for the ΔN protein of 13 kDa. Furthermore, all tested Orc1-2 domain mutants failed to show any up regulation upon NQO treatment, except for the two half DNA-binding site mutants, i.e., wH-m1 and wH-m2, which did show a level of up regulation in Orc1-2 comparable to the WT protein; nevertheless, the activation of TFB3 synthesis occurred only at a reduced level (Figure 2A). Thus, it appears the TFB3 induction level is more related to the drug sensitivity than Orc1-2 protein itself because the two half wH mutants are also more sensitive to NQO than WT (Table 1).

Next, reporter gene analysis was conducted for the gene promoters of *orc1-2*, *upsA*, and *cedA1* (Figure 2B). We found that the two-half binding site mutants, wH-m1 and wH-m2, that carrying mutations at R353R354 and R381R383, respectively, retained a residual NQO-responsive expression of ca. 40–45% and 10–15% of the WT for both *orc1-2* and *cedA1* genes, whereas DDR activation of these genes was completely abolished in all other mutants, including ΔN, ΔC, WA, ISM, and wH. Therefore, data from reporter gene assay may better reflect the DDR regulation of Orc1-2 expression than the data obtained from Western blot analysis.

Analysis of the expression of down-regulated DDR genes by Western blot analysis revealed that Vps4, Orc1-1, and Orc1-3 were down regulated in WT cells cultured in NQO-containing media (Figure 2A), and quantification of the corresponding protein bands revealed that the reduction at the protein level was 17–47%. In contrast, little change could be observed for any of the *orc1-2* mutants, including the mutants of the two half wH sites (Figure 2A). Nevertheless, RT-qPCR analysis of two cell division genes not only showed the drug-induced down regulation in WT, but slight down regulation was also observed for the two half wH mutants (Figure 2C). As expected, mutation of both predicted DNA-binding motifs in the wH domain eliminated DDR regulation, indicating that the wH domain of Orc1-2 plays a primary role in promoter binding (Figure 2). This conclusion is reinforced by the construction of the WBwH double mutant in which the Orc1-2E154A-induced cell death is suppressed by the deficiency of DNA binding (Table 1).

Therefore, we conclude that all three conserved domains are essential for the functionality of Orc1-2 in DDR regulation. Interestingly, mutation of each half wH DNA-binding site only yielded partial loss of the DDR regulation, suggesting that the Orc1-2 function can be modulated by changing its capability of interacting with promoter DNA. We inferred that Orc1-2-ATP could be the active form of the DDR regulator that facilitate cell cycle inhibition, and the regulation has to be offset by the hydrolysis of ATP to ADP, facilitated by the Walker B motif in the AAA+ ATPase. Thus, the Orc1-2E154A-driven cell lethality could be a consequence of the persistent activation of the expression of DDR genes that arrest the cell cycle progression.

### 2.5. Identification of Orc1-2 Domains Responsible for DNA Binding

**Figure 3:**
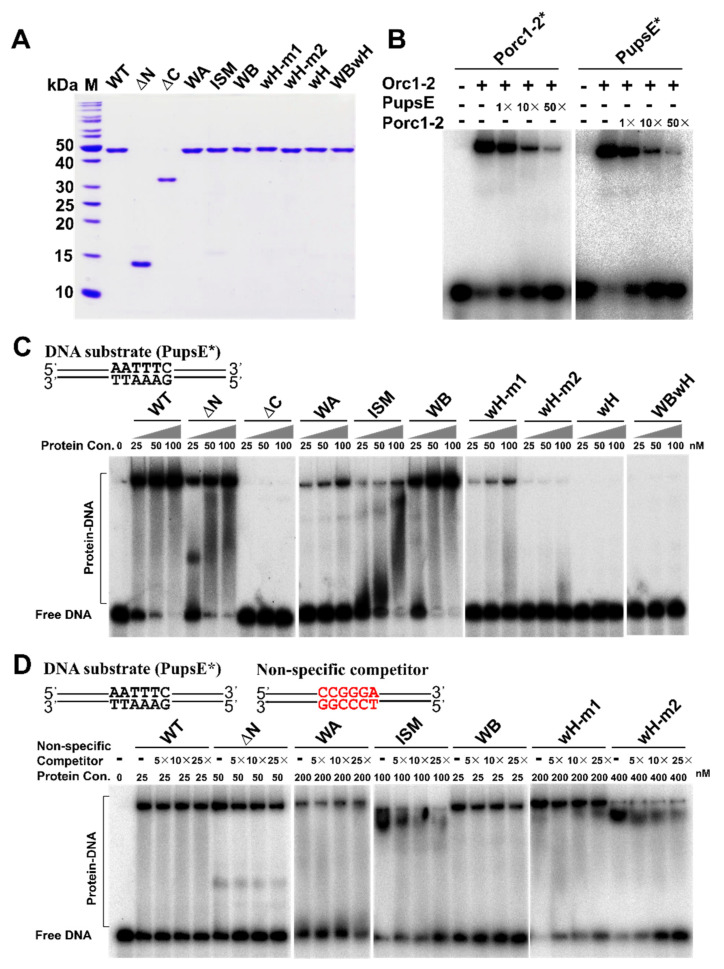
Orc1-2 affinity and specificity for the promoter of the DDR gene. (**A**) SDS-PAGE analysis of the wild-type Orc1-2 protein and its mutant derivatives purified from *E. coli*. ΔN, N-terminal domain (ATPase) truncation mutant; ΔC, C-terminal domain (wH) truncation mutant; WA, K72A at Walker A motif; ISM, G127DL128D at the initiator-specific motif (ISM); WB, E154A at Walker B motif; wH-m1, R353AR354A at the wH domain; wH-m2, R381AR383A at the wH domain; wH, quadruple substitutions at the wH domain; WBwH, pentuple substitutions at Walker B motif and wH domain. (**B**) EMSA assay of the wild-type Orc1-2 protein using the promoters of *orc1-2* and *upsE* genes. P_orc1-2_* and P_upsE_* DNA probes are of 46 bp promoter fragment containing 6 bp DDRE motif in the center and 20 bp flank region on each side. Binding assay was conducted with 25 nM Orc1-2 and ~2 nM radiolabeled probe (Porc1-2* or PupsE*) in the presence of the indicated amounts of unlabeled specific competitor. (**C**) Effects of mutation of each Orc1-2 domain on its interaction with the *upsE* promoter. DNA binding assays were performed with the indicated amounts of the WT or one of the mutated Orc1-2 proteins in the presence of ~2 nM radiolabeled PupsE* probe. (**D**) Determinants of the specific interaction between Orc1-2 and the promoter of *upsE*. DNA binding was conducted with an indicated amount of Orc1-2 or its mutant protein, ~2 nM radiolabeled PupsE*, and a specified amount of unspecific competitor DNA and analyzed by native PAGE.

To yield an insight into how Orc1-2 could interact with DDR promoters, the designed mutants were expressed in *Escherichia coli* and purified to apparent homogeneity (Figure 3A). The purified wild-type Orc1-2 and its mutated derivatives were then analyzed for their DNA binding activity.

Previous DNA foot-printing analyses revealed that Orc1-2 interacts with the ANTTTC hexanucleotide named DNA Damage Response Element (DDRE) motif on its own promoter [3], and the sequence motif is present on promoters of several DDR genes. A 46-bp promoter fragment (Table S1) was then designed for both *orc1-2* and *upsE* genes, the latter of which encodes a protein functioning in the synthesis of UV-induced pili in *S. islandicus*. Both promoter fragments carry the 6 bp DDRE motif in the center with 20 bp flanking sequence on each side. Testing the DNA binding of WT Orc1-2 to both promoters revealed that the regulator has very similar affinity to them both (Figure 3B). Then, P*_upsE_* was employed for comparing DNA binding between the WT Orc1-2 and its mutated variants using Electrophoretic Mobility Shift Assay (EMSA). The results are shown in Figure 3C. We found that ΔN formed large DNA–protein complexes, whereas ΔC retaining the entire AAA+ ATPase domain did not yield any DNA–protein complex, which is in full agreement with prediction of the wH domain as the DNA binding domain. Furthermore, mutation of each pair of Arg residues in the WH motifs strongly weakened the formation of protein–DNA complexes and substitution of all four Arg completely abolished the DNA binding (the wH mutation in Figure 3C). These results are consistent with the function of the wing-helix domain as a DNA binding motif in Orc1 proteins [32].

Nevertheless, while the ΔN truncation formed two distinct protein–DNA complexes at the lowest protein concentration tested here, the WT Orc1-2 readily formed a large protein–DNA complex at the same protein concentration (Figure 3C). These results suggested that the Orc1-2 N-terminal domain could also have a function in the protein–DNA interaction. Indeed, distinct properties were observed for AAA+ ATPase mutants: while ISM and Walker A mutations strongly reduced the formation of protein–DNA complexes, similar or even stronger DNA binding was observed for Walker B (Figure 3C). Because the Walker A mutation abolished the ATP binding of the protein whereas the Walker B mutation prevented the hydrolysis of ATP bound to the Walker A motif, these results indicated that, when complexed with ATP, Orc1-2 shows a strong binding activity to DDR promoters. Taken together, all three structural domains of Orc1-2 are important for DNA binding of the non-initiator.

Orc1-2 protein-promoter interactions exhibited additional features: complexes with ΔN and ISM mutant proteins were distributive (Figure 3C), suggesting there could be changes in the specificity between the mutated Orc1-2 protein and promoter interaction. To test this, a DNA fragment of a mutated *upsE* promoter carrying 5′-CCGGGA-3′, the transversion mutation of the DDRE motif (denoted as P*_upsE_*-mut), was used as a non-specific DNA in the EMSA assay. We found that the non-specific DNA significantly reduced the formation of promoter-Orc1-2 complexes with the ISM and wH-m2 mutants (Figure 3D). Thus, the G127L128 and R381R383 motifs in these two subdomains are responsible for the specific DNA–protein interaction between Orc1-2 and DDR promoters.

### 2.6. Orc1-2 Mediated Two Distinct Modes of Cell Death in S. islandicus

To yield a further insight into Orc1-2E154A-induced cell death, the *orc1-2* mutant gene was cloned to pSeSD, the *Saccharolobus* expression vector. The resulting expression plasmid pSe-*orc1-2^E152A^* was then transformed into Δ*orc1-2*, but no transformants were obtained in several consecutive attempts. Occasionally, one or a few colonies were obtained; analysis of plasmids present in the transformants revealed that the plasmid-borne *orc1-2* gene was reverted into the wild-type in all tested transformants. These transformation experiments reinforced the toxicity of the Orc1-2E154A protein.

To test if a low level of Orc1-2E154A could be tolerated in this archaeon, attempts were made to replace P*_araS-SD_* with the original P*_araS_* promoter, or P*_araS-50_* or P*_araS-38_*; the latter two are P*_araS_* derivatives [33]. We found that transformants were obtained with expression plasmids carrying the WT *orc1-2* gene fused to each of the above promoters, but for expression of the WB-deficient Orc1-2 protein, only the plasmid carrying the P*_araS-38_*-*orc1-2*^E154A^ fusion gene yielded transformants on the SCV plate. Because P*_araS-38_* is also an arabinose-inducible promoter, these strains were suitable for comparative investigations of the WT regulator and its E154A substitution mutant protein.

**Figure 4:**
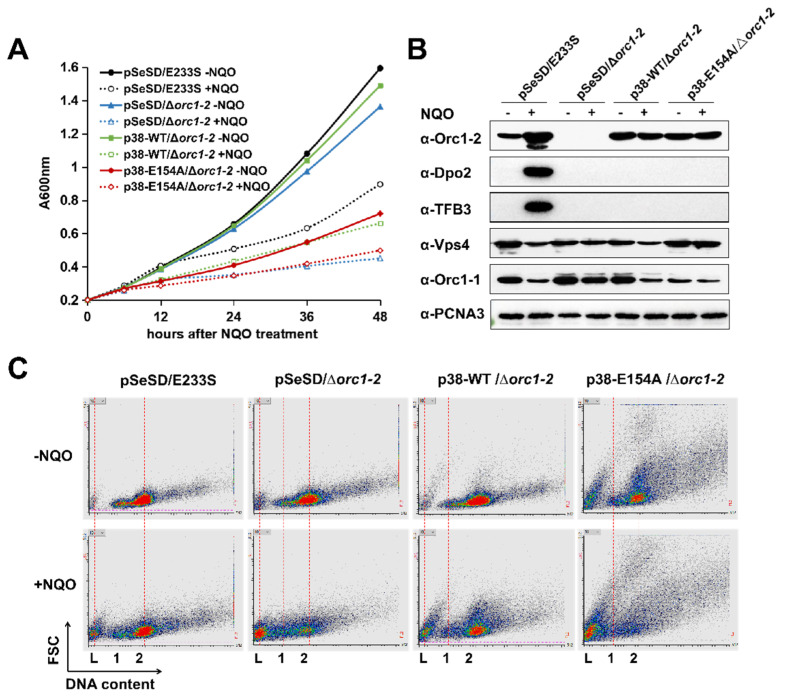
Expression of Orc1-2^E154A^ in *S. islandicus*. (**A**) Overexpression of Orc1-2E154A causes growth retardation in an NQO-independent manner. *Saccharolobus* cells were grown in ACV medium with or without 2 μM NQO (indicated as +NQO and −NQO, respectively). Cultures were incubated for 48 h, during which cell samples were taken for monitoring their A600 values. (**B**) The expression level of selected DDR proteins in each strain. Exponentially growing cultures (OD_600_ = 0.2) were treated by 2 μM NQO for 12 h, and 10 μg of total cell extracts of each sample was used for Western blot analysis. PCNA3 was used as the loading control protein. (**C**) Expression of Orc1-2E154A facilitates cell death. Flow cytometry profile of cell samples taken after 24 h NQO exposure. The results were shown in FL2 (DNA content, horizontal axis)-FSC (Forward scattered light, vertical axis) cytograms. Both axes are shown in liner scale. L: DNA-less cells; 1: cell population containing one chromosome; 2: cells containing two chromosomes.

Four strains, including p38-WT/Δ*orc1-2,* p38-E154A/Δ*orc1-2* and the two references, pSeSD/E233S and pSeSD/Δ*orc1-2*, were then chosen for further characterization. These strains grew similarly in SCV; upon DNA damage treatment, the strains carrying a p38 plasmid behaved like the Δ*orc1-2* strain (Figure S3), consistent with their low level of WT or E154A in the SCV cultures (Figure S4). In ACV, however, the growth of the strain p38-E154A/Δ*orc1-2* is already strongly inhibited, and the inhibition is NQO-independent; addition of NQO into the medium imposed severe growth inhibition to all four cultures (Figure 4A). Strikingly, the E154A mutation effect and the NQO inhibition effect are additive, and together they yielded a very similar extent of growth inhibition to that observed for Δ*orc1-2* (Figure 4A).

To test how the Orc1-2 level could be related to the phenotype, Orc1-2 or Orc1-2^E154A^ was analyzed by Western blot analysis. We found the regulator protein from plasmid-borne expression in ACV (driven by the P*_araS-38_* promoter) reached ca. 2-fold of the uninduced level expressed from the chromosomal locus in the WT strain; nevertheless, their expression was reduced to <1/8 when grown in SCV (Figure S3). These p38 strains were hypersensitive to the NQO in SCV, as for Δ*orc1-2* (Figure S4). More strikingly, the p38 strain expressing Orc1-2E154A exhibited anomalous morphologies regardless of DNA damage treatment (Figure S5). Taken together, these results indicated that: (a) Orc1-2^E154A^ is capable of mediating cell cycle arrest at a relatively low concentration and in the absence of any DNA damage signal; (b) the same amounts of the WT Orc1-2 can also inhibit cell growth upon DNA damage treatment. In both cases, cell death occurred, along with reduction in the ESCRT-III protein (Figure 4B), a representative protein involved in growth inhibition in *Saccharolobus*. Furthermore, upon NQO treatment, cell death is strongly facilitated in p38-E154A/Δ*orc1-2* cells (Figure 4C), and the additive nature of the two cell death events indicates that different cell death pathways are induced, i.e., one of which is induced by the wild-type Orc1-2 upon DNA damage treatment while the other is induced by the Orc1-2E154A; only the latter is characterized with the occurrence of anomalous cell morphologies.

The response of Orc1-2-activated genes was also investigated. Strains carrying a p38 plasmid failed to show DDR induction of TFB3 and Dpo2 synthesis (Figure 4B), which is consistent with the lack of cell aggregation for both cultures (Figure S5). The physiological traits of these strains are summarized in Table 2. Taken together, the Orc1-2 regulation occurs in at least two steps: the first involves the enforcement of growth inhibition and the second is the activation of expression of DNA repair proteins. This unique feature renders it possible for Orc1-2 to respond to different signals, thus functioning more generally in stress-responsive regulation in this archaeon.

## 3. Discussion

Our characterization of the structural domains of *S. islandicus* Orc1-2 has revealed that their functions in DDR are analogous to the functions of the conserved domains in Orc1-1 and Orc1-3, the replication initiators, but important differences also emerge in protein–DNA interactions and the functions of nucleotides and ATPase.

### 3.1. Distinct Features of Orc1-2 Protein–DNA Interactions

To date, the crystal structures of three crenarchaeal Orc1 proteins have been determined, including the monomeric origin DNA-Orc1 complex of *A. pernix* and the heterodimeric origin DNA-Orc1-Orc1-3 complex of *S. solfataricus.* In the reported structures, the Orc1 proteins interact with origin DNAs in different fashions. Common for them both is the wH domain interacting with ORB motifs of a dyad symmetry. However, while ISM is engaged in DNA binding in the monomer complex, interacting with the G-track motif in the origin via van der Waals interactions [25], the *S. solfataricus* origin (*oriC2*) carries two ORB sites: one for Orc1-1, the other for Orc1-3, and for this reason the two initiators can form a heterodimer on the origin element. However, in this case the ISM domain no longer interacts with the origin DNA [24]. To date, structures have not been determined for any promoter DNA-Orc1-2 protein. Nevertheless, the corresponding binding sequence of Orc1-2 in DDR promoters is predicted as the core element of DDRE with the sequence of ANTTTC, which is not symmetric. Indeed, our analysis showed that among the two pairs of arginine residues (R353, R354; R381, R383) interacting with the DDR promoter, only the pair of R381 and R383 is engaged in the specific DNA binding. At present, it is not known where the ISM domain binds onto DDR promoters; however, mutagenesis of this domain revealed that it is essential for the specific interaction between Orc1-2 and DDR promoters (Figure 3). Therefore, we have reasoned there must occur a fast evolution in the wH domain for Orc1-2 non-initiators during evolution in order to turn an initiator into a DDR regulator.

### 3.2. Functions of the AAA+ ATPase Domain

Orc1 proteins belong to the AAA^+^ ATPase subfamily. Investigation of the function of ATP in Orc1 initiators is often related to its function of recruitment of the MCM helicase during the initiation of DNA replication [8,31], a function that is irrelevant to Orc1-2, a non-initiator. Nevertheless, a crystal structure study revealed that the active site of the AAA+ ATPase is occupied by ADP [25], and the ADP-bound form of Orc1-1 from *A. pernix* has more conformational flexibility for the wH domain, and furthermore, ATP has the potential to lock the protein at a certain conformation [18]. This finding is intriguing because it provides the basis for the observation that ATP and DNA binding remodels the structure of Orc1 initiators [8]. Because Orc1-2E154A is locked constantly in the ATP-bound form and it mediates cell death, it is very likely that the remodeling of Orc1-2 by ATP and DNA-binding probably locks the regulator onto the gene promoters of DDR genes. As a consequence, the expression of these genes can no longer be switched on or off, and this could lead to cell death, as we have observed with p38-E154A/Δ*orc1-2* cells. This regulation is analogous to the results observed with the *Saccharolobus* Orc1-1E147A mutant that drives initiation of *oriC1* in vivo [8] and functions in the loading of the MCM helicase in vitro [31]. However, the Walker B mutation in Orc1-1 does not lead to any growth inhibition to this archaeon, which is in strict contrast to the same mutation in Orc1-2.

### 3.3. Orc1-2-Directed DDR Regulation Is a Stepwise Process

In this work, we have generated two unique genetic materials for investigation of Orc1-2 functions. These are Δ*orc1-2*, carrying either p38-WT or p38-E154A. Because their expression is controlled by the same promoter, both Orc1-2 proteins are expressed to a very similar level, which attained to ca. two-fold of non-induced Orc1-2, i.e., equivalent to ca. one fourth of the level in the induced strains (Figure S4). Investigation of these two strains has revealed two cell death events that have not been observed before: (a) the cell death pathway mediated by Orc1-2E154A is independent of DNA damage, and the process is also accompanied with the occurrence of anomalous cell morphologies, and (b) the cell death pathway conferred by the wild-type Orc1-2 in NQO can occur at ca. ¼ of the Orc1-2 level induced by DNA damage, as we observed in a previous work [3]. It would be very interesting to investigate how these two cell death events differ from each other.

Considering that the expression of the wild-type Orc1-2 to the same level or to a much higher level in our previous study did not reveal any growth delay or retardation in the absence of any DNA damage treatment [3], the observation of Orc1-2-induced cell death under the two unique conditions pinpoints stepwise regulation of Orc1-2 during DNA damage. In the first step, cell cycle progression is inhibited, and this occurs through the activation of Orc1-2 at a moderate level, such as the level produced from the plasmid-borne expression in the p38-WT strain. This step could also serve as a check point in cell cycle progression. If the extent of DNA damage is severe and extensive damage repair activity is required, Orc1-2 is then activated to the maximal induced level to activate expression of Dpo2, the error-prone DNA polymerase [34,35,36] and TFB3, the DDR coactivator [37], as well as proteins of the Ups and Ced systems, functioning in intercellular DNA transfer for DNA repair [38,39]. Further investigations are required to reveal which signal is responsible for the first step regulation and how the second regulation is enforced.

## 4. Materials and Methods

### 4.1. Cell Growth, Transformation and NQO Treatment of Saccharolobus

*S. islandicus* E233S carrying deletion alleles of Δ*pyrEF* and Δ*lacS* was constructed previously [40]. Δ*orc1-2* was reported recently [3]. Plasmids (Supplementary Table S1) were introduced into *Saccharolobus* strains by electroporation [41]. All *Saccharolobus* strains were cultured at 78 °C in SCV (basic salts plus 0.2% sucrose, 0.2% casamino acids, and 1%vitamin solution) or in ACV medium (in which sucrose is replaced with D-arabinose) [42], and uracil was added to 20 μg/mL if required.

DNA damage experiments of *S. islandicus* were conducted as previously described [43]. *Saccharolobus* cells were grown to an early growth phase (A_600_ = ca. 0.2), to which 4-nitroquinoline-1-oxide (NQO) was supplemented to 2 μM, and incubation was then continued. Cell samples were taken at different timepoints specified in each experiment and used for A600 measurement, microscopy, RNA extraction, β-glycosidase assay, Western blot analysis, and flow cytometry.

### 4.2. Construction of S. islandicus orc1-2 Mutants

The genome-editing plasmids (pGE) were constructed by following the strategy previously described [44]. Spacer fragments were generated by annealing of the corresponding complementary oligonucleotides (Supplementary Table S2) and inserted into pSe-Rp at the BspMI sites [45], yielding pAC plasmids carrying an artificial mini-CRISPR array. Then, donor DNA fragments carrying mutations of each target site in the *orc1-2* gene were obtained by SOE-PCR [46] using primers listed in Supplementary Table S2. The PCR products were digested with SalI and NotI and inserted into their cognate pAC plasmids (Supplementary Table S1) at the same sites, giving the pGE plasmids listed in Supplementary Table S1. Each pGE plasmid was introduced into *S. islandicus* E233S [40] by electroporation, giving transformants that were checked for the designed mutations by sequencing PCR products amplified from pGE transformants using checkF/checkR primers (Supplementary Table S2). Colonies carrying the designed *orc1-2* mutants were subjected to the *pyrEF* counter selection to cure the pGE plasmid. Verified mutants were used in further experiments.

### 4.3. Microscopy and Flow Cytometry

Cell morphologies and cell aggregations of *S. islandicus* cells were examined by the direct observation of fresh cultures under a Nikon Eclipse Ti-E inverted microscope (Nikon, Kobe, Japan). Data were obtained from at least 12 field-of-view images, and at least 500 cells were counted for each cell sample.

Flow cytometry of *S. islandicus* cells was conducted as previously described [47]. Briefly, the *Saccharolobus* cells were fixed with 70% ice-cold ethanol, stained with 40 µg/mL ethidium bromide (Sigma-Aldrich, St. Louis, MO, USA) and 100 µg/mL mithramycin A (Apollo Chemical, Tamworth, UK), and analyzed with an Apogee A40 cytometer (Apogeeflow, Hertfordshire, UK) equipped with a 405 nm laser. A dataset of at least 60,000 cells was collected for each sample.

### 4.4. Total RNA Preparation and Real-Time Quantitative PCR

*S. islandicus* cells were grown to A_600_ of ca. 0.20, to which 2 μM NQO was added. After cultivation for 6 h, cell mass was collected by centrifugation and employed for extraction of total RNAs using the Trizol reagent (Ambion, Austin, TX, USA), following the manufacturer’s procedure. Residual genomic DNA was removed from RNA preparations by treatment of DNase I (Thermo Scientific, Waltham, MA, USA). For quantitative reverse transcription-PCR (RT-qPCR), first-strand cDNAs were synthesized with the total RNA samples using an M-MuLV reverse transcriptase (Thermo-Scientific, Waltham, MA, USA) in the presence of random hexamer primers. The resulting cDNAs were used for quantitative PCR (qPCR) with Maxima SYBR Green/ROX qPCR Master Mix (2X) (Thermo Scientific, Waltham, MA, USA), using the primers listed in Table S2. PCR reactions were conducted with a CFX96 Touch^TM^ real-time PCR detection system (Bio-Rad, Hercules, CA, USA). The conditions of qPCR were as following: denaturing at 95 °C, 5 min; 40 cycles of 95 °C, 15 s; 55 °C, 15 s; and 72 °C, 20 s. Relative amounts of mRNAs of selected genes were calculated using the comparative Ct method [48] with 16S rRNA as a reference.

### 4.5. Western-Blotting Analysis

*S. islandicus* cells were cultured in SCV to an A_600_ of ca. 0.2, then NQO was added to 2 μM and incubation was continued for 6 h. Cell mass was then collected by centrifugation from each sample and re-suspended in 10 mM Tris-HCl buffer (pH = 8.0). Cell suspensions were sonicated to disrupt cells, and cellular extracts were obtained by removing cell debris by centrifugation at 13,000 rpm at 4 °C for 30 min. The protein concentrations of the soluble cell extracts were determined by using a Coomassie Protein Assay Kit (Thermo Scientific, Waltham, MA, USA), and similar amounts of the cell extracts (ca. 50 µg) were used for SDS–PAGE analysis to separate proteins according to their sizes. Then, fractionated proteins were transferred from the polyacrylamide gel onto a nitrocellulose blotting membrane (GE Healthcare, Waukesha, WI, USA), using the Semi-Dry Electrophoretic Transfer Cell system (Bio-Rad, Hercules, CA, USA). Western blot analyses of selected proteins in the samples were performed as described previously [37]. Briefly, the membrane was incubated in a 5% skim milk blocking agent for 2 h and was then incubated with individual primary antibodies raised against each protein (Orc1-2, TFB3, Vps4, Orc1-1, Orc1-2, and PCNA3) and finally with the horseradish peroxidase-labeled goat anti-rabbit antibody (Beyotime, Beijing, China). Protein bands were visualized using the ECL Western blot substrate (Thermo Scientific, Waltham, MA, USA) and visualized by exposure of the membrane to an X-ray film (Agfa HealthCare, Mortsel, Belgium).

### 4.6. Construction of Expression Plasmids of Mutated orc1-2 Genes and Purification of Their Encoded Proteins from E. coli

The truncation derivatives *orc1-2*Δ*N* (encoding for the C-terminal 115 amino acids) and *orc1-2*Δ*C* (encoding N-terminal 298 amino acids) were amplified from the genomic DNA of *S. islandicus* REY15A [7] by PCR using primer sets of orc1-2Cfwd-NdeI/orc1-2rev-NotI and orc1-2fwd-NdeI/orc1-2Nrev-NotI, respectively. Substitution mutants of *orc1-2* were constructed by SOE-PCR method [46]. Two overlapping primers were designed containing the desired mutations in the center of each pair of oligonucleotides, together with the primers orc1-2fwd-NdeI and orc1-2rev-NotI. The Orc1-2 wild type expression plasmid pET-orc1-2 constructed previously [3] was used as a template; all primers are listed in Supplementary Table S2. All mutated DNA fragments were inserted into pET-30a, giving pET-orc1-2ΔN, pET-orc1-2ΔC, pET-orc1-2WA, pET-orc1-2ISM, pET-orc1-2WB, pET-orc1-2WHM1, pET-orc1-2WHM2, pET-orc1-2WHM12, and pET-orc1-2WBWH (Supplementary Table S1). Those plasmids were then introduced into *E. coli* Rosetta cells.

WT and mutant Orc1-2 proteins were purified as described previously [3]. Briefly, *E. coli* cells containing different recombinant Orc1-2 expression plasmids were cultured in 500 mL LB medium containing 30 μg/mL kanamycin at 37 °C to the mid-log phase (A_600_ = ca. 0.6). IPTG was then added to 0.5 mM, and the culture was further cultured at 16 °C for 12 h. Cell mass was harvested by centrifugation and treated with a French press at 4 °C, and the resulting cell lysis was subjected to heat treatment at 70 °C for 5 min. After removing cell debris by centrifugation, the supernatant was loaded onto a 1 mL Ni-NTA column (GE Healthcare, Chicago, IL, USA) and His-tagged recombinant Orc1-2 protein was purified following the manufacturer’s instruction. Purified proteins were analyzed by SDS-PAGE, with their concentrations measured using a Coomassie Protein Assay Kit (Thermo Scientific, Waltham, MA, USA).

### 4.7. Electrophoretic Mobility Shift Assay

Two 46-bp DNA sequences were identified from the promoter region of the *orc1-2* gene and the *upsE* gene, giving P*_orc1-2_* and P*_upsE_*, each containing a 6 nt conserved DDRE motif (5-ANTTTC-3) in the center and two 20 nt flank regions on each side. Their DNA fragments were generated by the direct annealing of two complementary oligonucleotides followed by polyacrylamide gel electrophoresis (PAGE) purification. Both DNA fragments were 5′-labeled with 32p-γ-ATP (PerkinElmer, Waltham, MA, USA) using T4 polynucleotide kinase (New England Biolabs, Ipswich, MA, USA), and employed as DNA probes for electrophoretic mobility shift assay (EMSA). Specific competitor DNAs were the un-labelled DNA probes individually, whereas unspecific competitor DNAs carried the mutated DDRE motif. Recombinant Orc1-2 proteins were purified from *E. coli*, and EMSA experiments were conducted by mixing 2 nM labelled probe with an Orc1-2 protein (wt or mutant) at the concentration indicated in each experiment in the reaction buffer (20 mM HEPES (pH 7.6), 10 mM (NH_4_)_2_SO_4_, 1 mM DTT, 0.2% Tween-20, 30 mM KCl). Specific and un-specific competitors were added in the amounts indicated in the figures. The incubation was performed at 40 °C for 20 min. Samples were then loaded onto 8% native polyacrylamide gel using 40 mM Tris, 20 mM boric acid as the running buffer. After electrophoresis, the gels were scanned with a Typhoon FLA 7000 (GE Healthcare, Chicago, IL, USA).

## Figures and Tables

**Table 1:** Genetic analysis of structural domains of the *S. islandicus orc1-2* gene.

Name	Mutation	Mutant Viability	Protein Stability	NQO Sensitivity	Cell Aggregation	Cell Population (%)	
						<1	2
WT	413 aa	Yes	++	S	++	14.9	69.3
Δ*orc1-2*	In-frame deletion retaining 35 aa at N-terminus	Yes	NV	HS	−	72.0	20.0
ΔC	298 aa lacking wH domain	Yes	+	HS	−	75.0	15.5
ΔN	115 aa lacking ATPase domain	Yes	++	HS	−	77.8	8.9
WA	K72A Walker A	Yes	+	HS	−	69.4	22.5
WB	E154A WalkerB	No	++	NV	NV	NV	NV
ISM	G127D, L128D initiator specific motif	Yes	++	HS	−	73.3	19.7
wH-m1	R353A, R354A wH domain	Yes	++	S*	+	52.4	41.4
wH-m2	R381A, R383A wH domain	Yes	++	HS	−	73.7	18.7
wH	quadruple substitutions wH domain	Yes	++	HS	−	80.2	12.3
WBwH	E154A, R353A, R354A, R381A and R383A pentuple substitutions	Yes	++	HS	−	62.1	28.6

++: equivalent to WT; +: weaker than WT; −: little or no. NV: not available. S: sensitive; HS: hypersensitive; S*: moderately sensitive (between S and HS). Cell populations were quantified from flow cytometry data 48 h after addition of NQO. <1: DNA-less cells (dying cell population); 2: cells containing two chromosomes (live/dormant cell population).

**Table 2:** Comparative study of *S. islandicus* Strains Expressing WT Orc1-2 and Orc1-2E154A.

Name	Description	Promotor	Orc1-2 Level	Broth Growth		NQO Sensitivity	Cell Aggregation	Giant Cells
				SCV	ACV			
pSeSD/E233S	Genomic: Orc1-2	P*_orc1-2_*	++	++	++	S	++	No
pSeSD/Δ*orc1-2*	Orc1-2-deficient	NV	NV	++	++	HS	−	No
p38-WT/Δ*orc1-2*	Plasmid-borne: Orc1-2	P*_araS-38_*	+	++	++	HS	−	No
p38-E154A/Δ*orc1-2*	Plasmid-borne: Orc1-2E154A	P*_araS-38_*	+	++	+	HS	−	Yes

++: equivalent to WT; +: weaker than WT; −: little or no. NV: not available. S: sensitive; HS: hypersensitive.

## Data Availability

Not applicable.

## Supplementary Materials

The following supporting information can be downloaded at: https://www.mdpi.com/article/10.3390/ijms232314609/s1. References [8,40,45,49] are cited in the Supplementary Materials.
