# Supplementary material for: Dissection of Functional Domains of Orc1-2, the Archaeal Global DNA Damage-Responsive Regulator
Source: Int J Mol Sci. 2022 Nov 23;23(23):14609. doi: 10.3390/ijms232314609 (PMC9738581; doi:10.3390/ijms232314609)
Supplement: Supplementary file 1 [file ijms-23-14609-s001.zip › ijms-2020475-supplementary.pdf]

Supplementary Material

1. Supplementary Tables

Supplementary Table S1. Strains and plasmids used in this study

Strains	Genotype	Source
E233S	Δ*pyrEF*Δ*lacS*	Deng *et al*., 2009 [1]
Δ*orc1-2*	Δ*pyrEF*Δ*lacS*Δ*orc1-2*	Samson *et al*., 2013 [2]
ΔN	Δ*pyrEF*Δ*lacS*, carrying the deletion of Orc1-2 N-terminus ATPase domain (1-298 amino acids) coding sequence	This work
ΔC	Δ*pyrEF*Δ*lacS*, carrying the deletion of Orc1-2 C-terminus WH domain (299-413 amino acids) coding sequence	This work
WA	Δ*pyrEF*Δ*lacS*, carrying K72A mutation in the *orc1-2* gene	This work
ISM	Δ*pyrEF*Δ*lacS*, carrying G127D and L128D mutations in the *orc1-2* gene	This work
wH-m1	Δ*pyrEF*Δ*lacS*, carrying R353A and R354A mutations in the *orc1-2* gene	This work
wH-m2	Δ*pyrEF*Δ*lacS*, carrying R381A and R383A mutations in the *orc1-2* gene	This work
wH	Δ*pyrEF*Δ*lacS*, carrying R353A, R354A, R381A and R383A quadruple mutations in the *orc1-2* gene	This work
WBwH	Δ*pyrEF*Δ*lacS*, carrying E154A, R353A, R354A, R381A and R383A pentuple substitutions in the *orc1-2* gene	This work

**Plasmids**	**Features**	
pSe-Rp	The plasmid contains a DNA fragment of two tandem copies of CRISPR repeat sequences for the construction of the artificial mini-CRISPR loci	Peng *et al*., 2015 [3]
pSeSD	A *Saccharolobus-E. coli* shuttle vector carrying an expression cassette controlled under a synthetic strong promoter ParaS-SD	Peng *et al*., 2012 [4]
pAC-*orc1-2*ΔN, ΔC, WA, ISM, wH-m1, wH-m2, wH, WBwH	pSe-Rp carrying a spacer matching to the protospacer in the corresponding mutation site of *orc1-2* gene in genome	This work
pGE-*orc1-2*ΔN, ΔC, WA, ISM, wH-m1, wH-m2, wH, WBwH	The genome-editing plasmid derived from pAC-*orc1-2*ΔN, ΔC, WA, ISM, WHM1, WHM2, WHD, WB-WHD respectively, with the corresponding donor DNA inserted between SalI and NotI	This work
P38-WT	Derived from pSeSD, carrying Orc1-2 coding sequence which is controlled under a weak promoter ParaS38 [5]	This work
P38-E154A	Derived from pSeSD, carrying Orc1-2 E154A mutant coding sequence which is controlled under a weak promoter ParaS38	This work

Supplementary Table S2. Oligos used in this study

**Oligos**	**Sequence***
**Construction of *orc1-2* mutants**	
ΔN-SpF	AAAGTTCTATTTATAAATTAGATAGAAGTATAAGGGATCATATA
ΔN-SpR	TAGCTATATGATCCCTTATACTTCTATCTAATTTATAAATAGAA
ΔN-SalIF	ACGCGTCGACCCATTTCAATCGATTATA
ΔN-NotIR	ATAAGAATGCGGCCGCATAATATCTTCATTTTAG
ΔN-SOEF	GATTGGGGTAAAAGTTATGATTCAAGAGATTGTGGAT
ΔN-SOER	ATCCACAATCTCTTGAATCATAACTTTTACCCCAATC
ΔN-checkF	ACCCATTAGTAATTTATGGTCT
ΔC-SpF	AAAGTAGAAGAGGAATATATATCATTAGCCAGAGAGTTTAATGA
ΔC-SpR	TAGCTCATTAAACTCTCTGGCTAATGATATATATTCCTCTTCTA
ΔC-SalIF	ACGCGTCGACACTTCTTAGTAAGATTGT
ΔC-NotIR	ATAAGAATGCGGCCGCGCAATCACGTTCCCCTTCTC
ΔC-SOEF	TACAATAAATCCAGAGTAATCTATAATTCATCTAAAA
ΔC-SOER	TTTTAGATGAATTATAGATTACTCTGGATTTATTGTA
WA-SpF	AAAGTTTTCCCAGTACCTGTCCTACCTACTATTACAACTCTTTC
WA-SpR	TAGCGAAAGAGTTGTAATAGTAGGTAGGACAGGTACTGGGAAAA
WA-SalIF	ACGCGTCGACCCATTTCAATCGATTATA
WA-NotIR	ATAAGAATGCGGCCGCGCATTACCATTACCCCCT

WA-SOEF	ACTGGAGCCACCGCAACTGTTAGATTGTTT
WA-SOER	AGTTGCGGTGGCTCCAGTACCTGTCCTACCTAC
WA-checkR	ATCTAACAGTTGCGGTGGCT
WB-SpF	AAAG ATCTAATGCAACTATTAGATGTATATTTCTCCTATCTAAA
WB-SpR	TAGCTTTAGATAGGAGAAATATACATCTAATAGTTGCATTAGAT
WB-SalIF	ACGCGTCGACCCATTTCAATCGATTATA
WB-NotIR	ATAAGAATGCGGCCGCGCATTACCATTACCCCCT
WB-SOEF	TTGGACGCTTTTGGACATTTCCTGAAT
WB-SOER	ATGTCCAAAAGCGTCCAATGCAACTATTAGATGTAT
WB-checkF	CTAATAGTTGCATTGGACGCT
ISM-SpF	AAAGAGGGGATTATCAGCACAAGAAGTATTTAAGATTATCCATG
ISM-SpR	TAGCCATGGATAATCTTAAATACTTCTTGTGCTGATAATCCCCT
ISM-SalIF	ACGCGTCGACAAGGATATACTTTCAGAT
ISM-NotIR	ATAAGAATGCGGCCGCGCATTACCATTACCCCCT
ISM-SOEF	CCGAGCCGAGACGACTCAGCACAAGAAGTATTTAAGAT
ISM-SOER	TGAGTCGTCTCGGCTCGGTATTGGTAGTTTTAACGCGT
ISM-checkF	CCAATACCGAGCCGAGACGAC
wHm1-SpF	AAAGTCTAGGTTCCTCATTAAACTCTCTGGCTAATGATATATAT
wHm1-SpR	TAGCATATATATCATTAGCCAGAGAGTTTAATGAGGAACCTAGA
wHm1-SalIF	ACGCGTCGACACTTCTTAGTAAGATTGT

wHm1-NotIR	ATAAGAATGCGGCCGCGCAATCACGTTCCCCTTCTC
wHm1-SOEF	GAACCTGCTGCTCATACACAAGTTTATGAGTATCTG
wHm1-SOER	TGTATGAGCAGCAGGTTCCTCATTAAACTCTCTGGC
wHm1-checkR	TAAACTTGTGTATGAGCAGC
wHm2-SpF	AAAGTCCTCTCATTCCCTTCCCACTCTGCCTAGTATTAATTATC
wHm2-SpR	TAGCGATAATTAATACTAGGCAGAGTGGGAAGGGAATGAGAGGA
wHm2-SalIF	ACGCGTCGACACTTCTTAGTAAGATTGT
wHm2-NotIR	ATAAGAATGCGGCCGCGCAATCACGTTCCCCTTCTC
wHm2-SOEF	GGAATGGCTGGAGCTACGACTCTAGTTTCCCTTTCT
wHm2-SOER	AGTCGTAGCTCCAGCCATTCCCTTCCCACTCTGCCT
wHm2-checkR	ACTAGAGTCGTAGCTCCAGC
**Overexpression of Orc1-2**	
Orc1-2-NdeIF	GAAGGAGATATACATATGTTGGTTTCAGCTAAGGATAT
Orc1-2-NotIR	GTGCTCGAGTGCGGCCGCAGCCTTCGATTTTAACCTCA
Orc1-2ΔN-NdeIF	GGTAAAACATATGATTCAAGAGATTGTGGAT
Orc1-2ΔC-NotIR	ATTGCGGCCGCCTCTGGATTTATTGTAGA
Orc1-2WA-SOEF	ACAGGTACTGGGGCAACAGCAACTGT
Orc1-2WA-SOER	ACAGTTGCTGTTGCCCCAGTACCTGT
Orc1-2WB-SOEF	GTTGCATTAGATGCGTTTGGACATTTC
Orc1-2WB-SOER	GAAATGTCCAAACGCATCTAATGCAAC

Orc1-2ISM-SOEF	CCGTCGAGGGACGACTCAGCACAAGAAGTATTTAAGAT
Orc1-2ISM-SOER	TGAGTCGTCCCTCGACGGTATTGGTAGTTTTAACGCGT
Orc1-2wHm1-SOEF	GAACCTGCCGCCCATACACAAGTTTATGAGTATCTG
Orc1-2wHm1-SOER	TGTATGGGCGGCAGGTTCCTCATTAAACTCTCTGGC
Orc1-2wHm2-SOEF	GGAATGGCCGGAGCCACGACTCTAGTTTCCCTTTCT
Orc1-2wHm2-SOER	AGTCGTGGCTCCGGCCATTCCCTTCCCACTCTGCCT
**EMSA**	
orc1-2pF	TTTGAGAAAGAATCAAGAAAAGTTTCGGACTACTCTCTTAGATAAA
orc1-2pR	TTTATCTAAGAGAGTAGTCCGAAACTTTTCTTGATTCTTTCTCAAA
upsEpF	CTATAAAGGAAGTTTTAAGTAATTTCGGTATTGTGTGAGTATATTT
upsEpR	AAATATACTCACACAATACCGAAATTACTTAAAACTTCCTTTATAG
upsEpmutF	CTATAAAGGAAGTTTTAAGTCCGGGAGGTATTGTGTGAGTATATTT
upsEpmutR	AAATATACTCACACAATACCTCCCGGACTTAAAACTTCCTTTATAG

***The restriction sites are underlined and the DNA damage response elements (DDRE) are double-underlined.**

2. Supplementary Figures

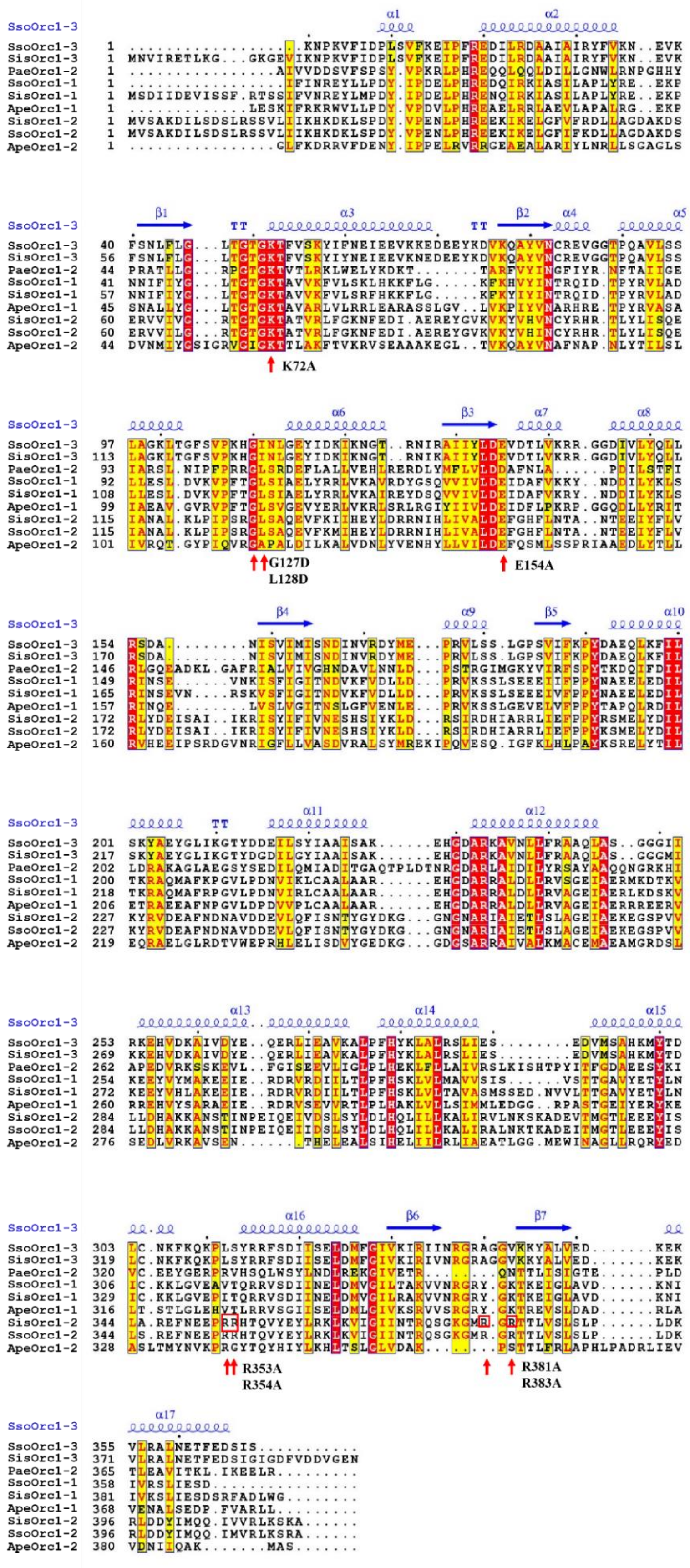

Supplementary Figure S1. Structure-based sequence alignments of selected ORC1 proteins.

Both Orc1 initiators (Orc1-1, Orc1-3) and Orc1 non-initiators (Orc1-2) proteins are included. Orc1 proteins are named with abbreviations of their names of genus and species, which are followed by their Orc name. Sso, *Saccharolobus solfataricus*; Sis, *Saccharolobus islandicus*; Pae, *Pyrobaculum aerophilum*; Ape, *Aeropyrum pernix*. The different secondary structure elements shown are alpha helices as large squiggles labelled (α), beta strands as arrows (β), and beta turns (TT). Identical residues are shown in white on red background, and conserved residues in red. The representative Orc1 structures are SsoOrc1-3. This figure was generated with ESPript.

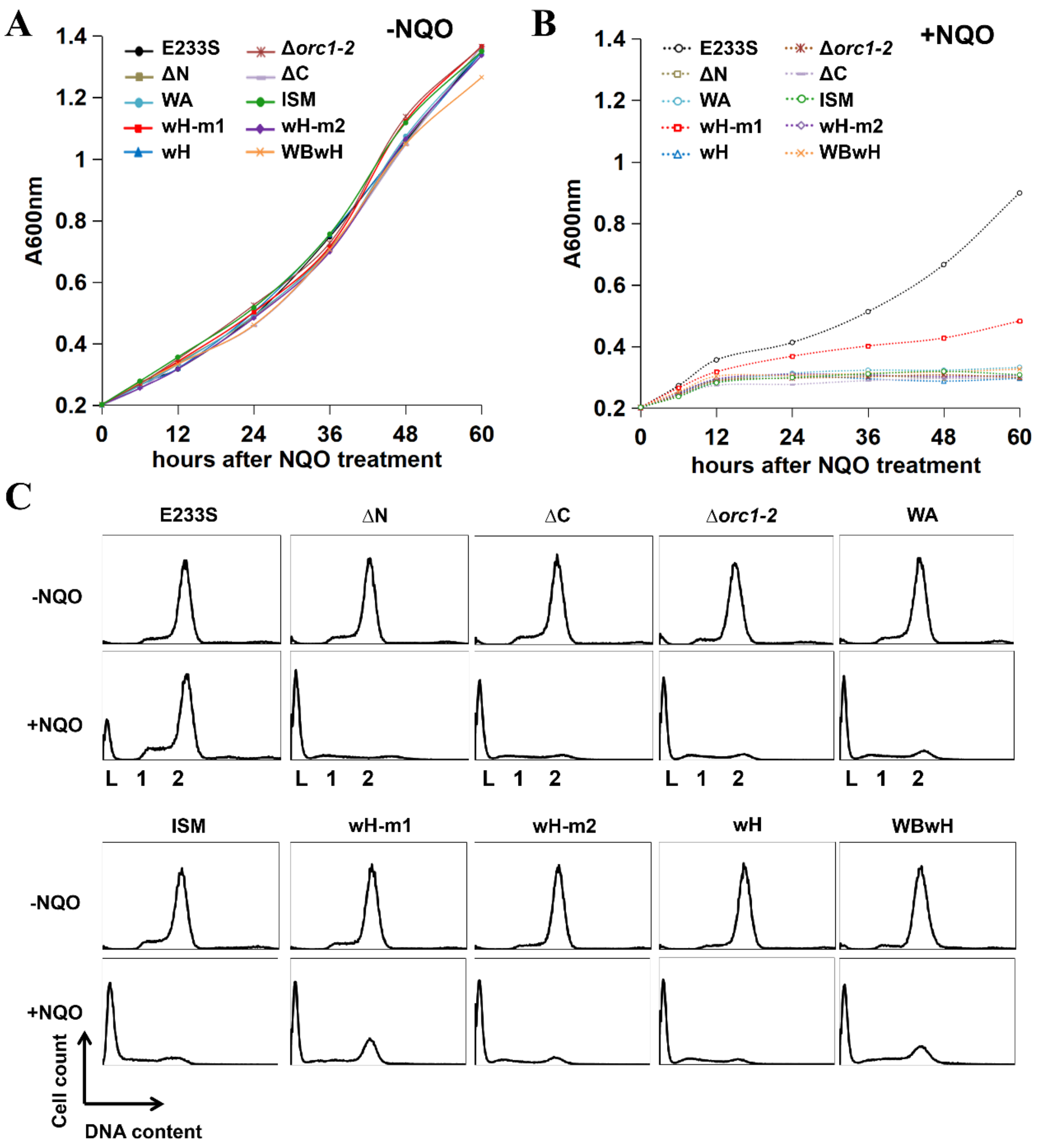

Supplementary Figure S2. Physiological characterization of *orc1-2* mutants

(A) and (B) Growth curves of E233S and *orc1-2* mutants. *Saccharolobus* cells were inoculated to SCV media in the absence (A) or presence (B) of 2 μM NQO, and grown for 60 h. Culture growth was estimated by measuring A600 values of individual cultures during incubation, with obtained values plotted against the incubation time. (C) Flow cytometry profile of cell samples taken at 48 h after NQO addition. DNA contents were divided into 256 arbitrary points on the X-axis, and plotted against the cell counts (Y-axis). L: DNA-less cells; 1: cells containing one chromosome; 2: cells containing two chromosomes.

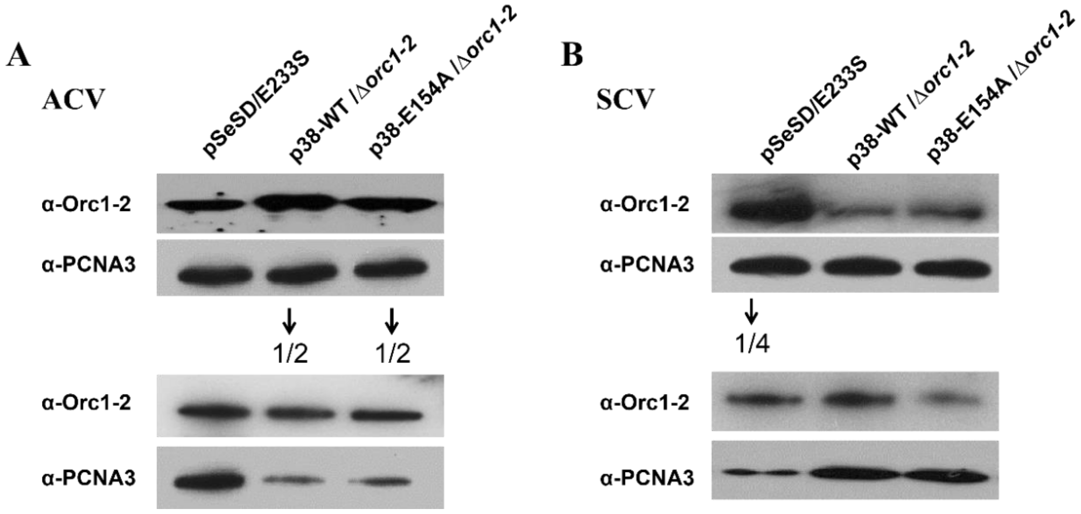

Supplementary Figure S3. Semi-quantification of Orc1-2 protein expressed from $P_{araS\text{-}38}$ promoter

Saccharolobus cells were grown in SCV or ACV medium. Cell mass was collected from which total proteins were prepared and used for western blot analysis. Some cell extract samples were also diluted for 2 (ACV) or 4 (SCV) folds before analysis. PCNA3 (one of the subunits of the replication clamp) was used as the loading reference.

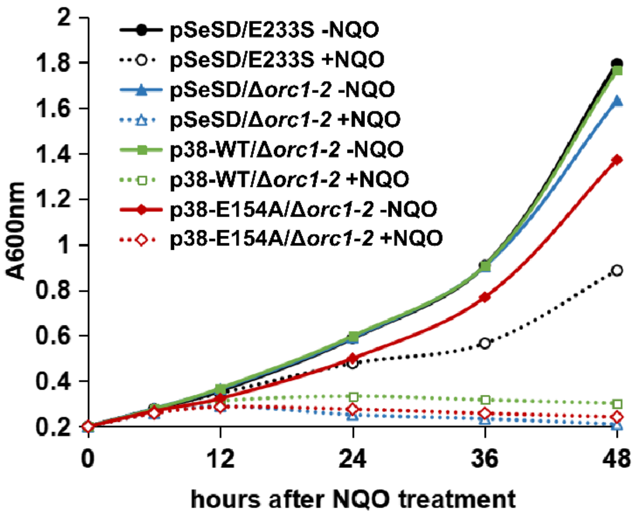

Supplementary Figure S4. Effects of E154A mutation on growth of *S. islandicus*

Cell growth was evaluated by determination of absorbance at 600nm. *Saccharolobus* cells were grown in SCV medium with or without 2 μM NQO for 48 h during which cell samples were taken for monitoring their OD_{600} values.

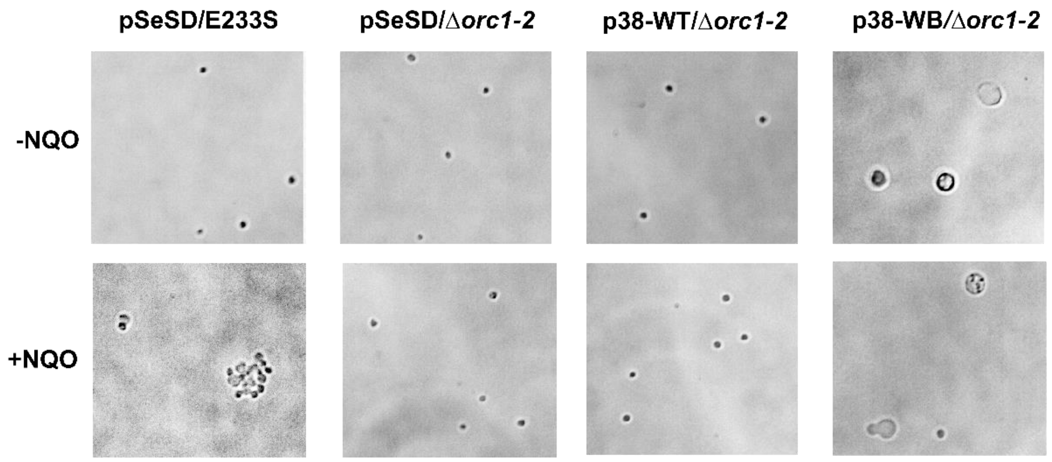

Supplementary Figure S5. E154A mutation of Orc1-2 facilitates formation of anomalous cells of *S. islandicus*.

Saccharolobus cells were grown in ACV medium with or without 2 μM NQO for 12 hours. Fresh culture samples were directly observed under a microscope.

1. Deng, L.; Zhu, H.; Chen, Z.; Liang, Y. X.; She, Q., Unmarked gene deletion and host–vector system for the hyperthermophilic crenarchaeon *Sulfolobus islandicus*. *Extremophiles* **2009,** 13, (4), 735-746.
2. Samson, R. Y.; Xu, Y.; Gadelha, C.; Stone, T. A.; Faqiri, J. N.; Li, D.; Qin, N.; Pu, F.; Liang, Y. X.; She, Q., Specificity and function of archaeal DNA replication initiator proteins. *Cell reports* **2013,** 3, (2), 485-496.
3. Peng, W.; Feng, M.; Feng, X.; Liang, Y. X.; She, Q., An archaeal CRISPR type III-B system exhibiting distinctive RNA targeting features and mediating dual RNA and DNA interference. *Nucleic acids research* **2015,** 43, (1), 406-417.
4. Peng, N.; Deng, L.; Mei, Y.; Jiang, D.; Hu, Y.; Awayez, M.; Liang, Y.; She, Q., A synthetic arabinose-inducible promoter confers high levels of recombinant protein expression in hyperthermophilic archaeon Sulfolobus islandicus. *Applied and environmental microbiology* **2012,** 78, (16), 5630-5637.
5. Peng, N.; Xia, Q.; Chen, Z.; Liang, Y. X.; She, Q., An upstream activation element exerting differential transcriptional activation on an archaeal promoter. *Molecular microbiology* **2009,** 74, (4), 928-939.
